# Correction to: M2 Macrophagy-derived exosomal miRNA-5106 induces bone mesenchymal stem cells towards osteoblastic fate by targeting salt-inducible kinase 2 and 3

**DOI:** 10.1186/s12951-021-00828-1

**Published:** 2021-03-27

**Authors:** Yuan Xiong, Lang Chen, Chenchen Yan, Wu Zhou, Tao Yu, Yun Sun, Faqi Cao, Hang Xue, Yiqiang Hu, Dong Chen, Bobin Mi, Guohui Liu

**Affiliations:** 1 Department of Orthopaedics, Union Hospital, Tongji Medical College, Huazhong University of Science and Technology, Wuhan, 430022 China; 2 Department of Orthopedic Surgery, Tongji Hospital, Tongji University School of Medicine, Shanghai, 200065 China; 3 Department of Neurosurgery, Union Hospital, Tongji Medical College, Huazhong University of Science and Technology, Wuhan, 430022 China

## Correction to: J Nanobiotechnol (2020) 18:66 https://doi.org/10.1186/s12951-020-00622-5

Following publication of the original article [1], the authors reported that Fig. 2 was not updated during the production process.

The updated Fig. 2 is provided below and the original article [1] has been corrected.

**Fig. 2:**
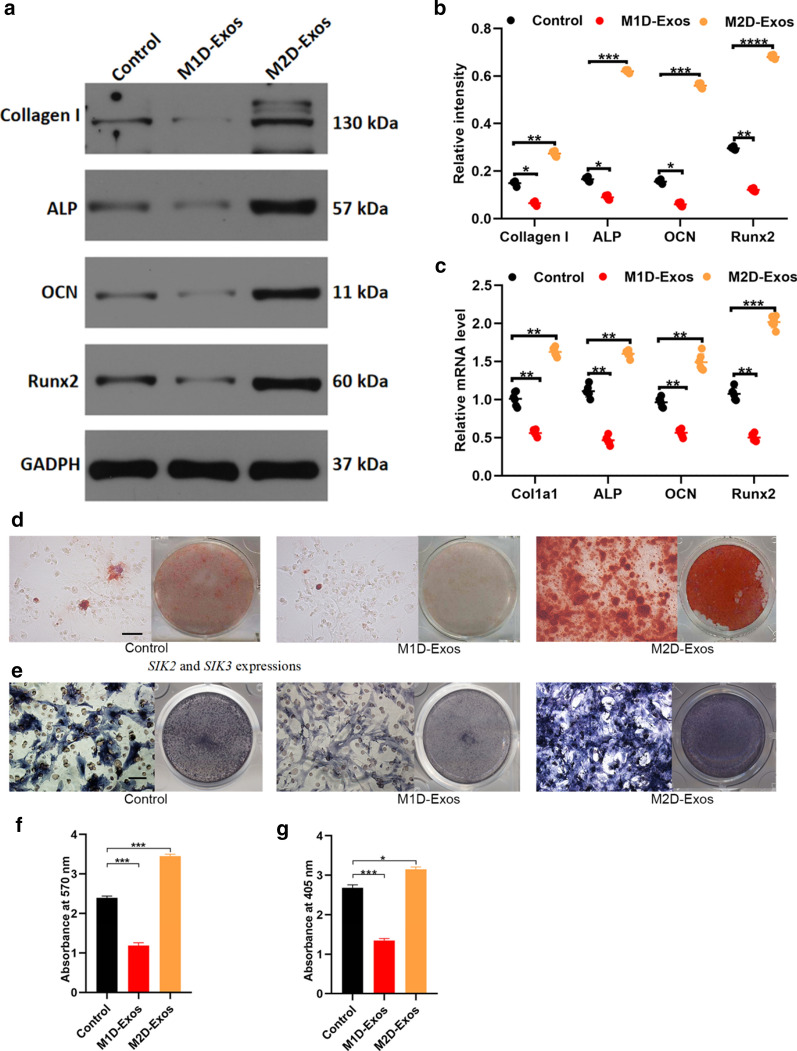
M2D-Exos induce osteoblast activity and matrix mineralization. **a** Osteogenic genes were upregulated in M2D-Exos-treated BMSCs measured by western blotting analysis; **b** The relative intensity of western blotting analysis; **c** Overexpression of the four osteognic genes can be detected in M2D-Exos groups measured by qRT-PCR analysis; **d** Alizarin red-mediated calcium staining in BMSCs following treated by PBS (control group), M1D-Exos, and M2D-Exos for 21 days. Scale bar = 10 mm; **e** ALP staining in BMSCs following treated by PBS (control group), M1D-Exos, and M2D-Exos for 14 days. Scale bar = 10 mm; **f**, **g** The statistical data of Alizarin red-mediated calcium staining and ALP staining. Data are mean ± SD of triplicate experiments. *p < 0.001, **p < 0.01, ***p < 0.001
